# Mechanistic insights into cardiac regeneration and protection through MEIS inhibition

**DOI:** 10.55730/1300-0152.2716

**Published:** 2024-10-30

**Authors:** Aynura MAMMADOVA, Galip Servet ASLAN, Li MO, Qifang WU, Sebastian CLAUSS, Fatih KOCABAŞ

**Affiliations:** 1 Regenerative Nanomedicine, INSERM-University of Strasbourg, Strasbourg, France; 2 Department of Genetics and Bioengineering, Faculty of Engineering, Yeditepe University, İstanbul, Turkiye; 3 Institute for Cardiovascular Regeneration, Goethe University, Frankfurt, Germany; 4 Department of Medicine I, University Hospital, LMU Munich, Munich, Germany; 5 German Center for Cardiovascular Research (DZHK), Partner Site Munich, Munich Heart Alliance, Munich, Germany; 6 Institute of Surgical Research at the Walter-Brendel-Centre of Experimental Medicine, University Hospital, LMU Munich, Munich, Germany; 7 Interfaculty Center for Endocrine and Cardiovascular Disease Network Modelling and Clinical Transfer (ICONLMU), LMU Munich, Munich, Germany

**Keywords:** MEIS1, cardiac regeneration, cardiac protection, cardiomyocyte proliferation

## Abstract

MEIS1, a member of the TALE-type homeobox gene family, has emerged as a pivotal regulator of cardiomyocyte cell cycle arrest and represents a promising therapeutic target. Our study reveals that inhibition of MEIS1 using two novel small molecules, MEISi-1 and MEISi-2, significantly enhances neonatal cardiomyocyte proliferation and cytokinesis. Specifically, MEISi-1 and MEISi-2 increased the proportion of proliferating cardiomyocytes (Ph3+TnnT cells) up to 4.5-fold and the percentage of cytokinetic cardiomyocytes (AuroraB+TnnT cells) by 2-fold, compared to untreated controls. MEIS1 inhibition resulted in a notable downregulation of MEIS1 target genes and cyclin-dependent kinase inhibitors, demonstrating its effect on key regulatory pathways. Additionally, the culture and differentiation of human induced pluripotent stem cells into cardiomyocytes were studied, with MEIS1 inhibition showing no adverse effects on cell viability. Extended treatment with MEIS inhibitors led to a substantial upregulation of critical cardiac-specific genes, including a 15-fold increase in the expression of Nkx2.5. This upregulation significantly impacted both cardiac mesoderm and cardiac progenitor cells. These findings underscore the potential of MEIS1 inhibitors as effective agents in enhancing cardiac regeneration and highlight their therapeutic promise in regenerative cardiology.

## 1. Introduction

MEIS genes, part of the TALE superclass of homeodomain proteins, are transcription factors crucial for diverse biological processes, such as development and tissue-specific gene expression (Moskow et al., 1995; Chang et al., 1997; Laurent et al., 2008; Mallo and Alonso, 2013). The MEIS gene family includes members like Meis1, Meis2, and Meis3 with multiple isoforms through alternative splicing (Moskow et al., 1995; Dibner et al., 2001; Zhang et al., 2002; Bhanvadia et al., 2018). In recent years, there has been a growing acknowledgment of the crucial role played by MEIS proteins and their associated counterparts in a wide array of biological processes, spanning regeneration (Jiang et al., 2021), stem cell functionality (Walasek et al., 2012; Miller et al., 2016), cellular metabolism (Kocabas et al., 2012), tumor development (Imamura et al., 2002; Ferreira et al., 2016; Burstin et al., 2017; Wang and Ghareeb, 2019; Meriç and Kocabas, 2022), and modulation of lifespan. Notably, MEIS1 is essential for cardiac development and hematopoiesis (Aksoz et al., 2018; Paul et al., 2019), MEIS2 plays key roles in limb and eye development with relevance to neurodevelopmental disorders (Liu et al., 2020; Delgado et al., 2021), and MEIS3, while less explored, contributes to specific developmental contexts (Dibner et al., 2001). MEIS proteins play essential roles in critical pathways like HOX (VanOpstall et al., 2020), Wnt (Arata et al., 2006), and Hedgehog signaling (Fabik et al., 2020), exerting influence over body segment identity, limb development, neural tube formation (Fabik et al., 2020; Liu et al., 2020), heart development (Machon et al., 2015; Aksoz et al., 2018; Paul et al., 2019), and eye development (Liu et al., 2020). Grasping the multifaceted functions of MEIS proteins in these pathways and their therapeutic modulation is of substantial relevance for the field of regenerative medicine and provides valuable perspectives into the underlying causes of developmental abnormalities and congenital disorders.

Our recent research has been dedicated to developing MEIS1 inhibitors, capitalizing on our specialized tools and expertise in MEIS1 biology (Arkin and Wells, 2004; Turan and Kocabas, 2020; Girgin and Kocabas, 2023; Meriç et al., 2023). Two recently identified MEIS1 small-molecule inhibitors, namely MEISi-1 and MEISi-2, have demonstrated the ability to enter cells and produce dose-dependent effects. Importantly, they operate by disrupting the interaction between the MEIS1 homeodomain and target DNA, thereby impairing the activation of MEIS1-targeted gene expression, including Hif-1α, Hif-2α, and p21. Furthermore, they show promise in expanding and enhancing the self-renewal potential of human and murine hematopoietic stem cells in vitro and in vivo. In addition, recent knockout studies in animal models showed that the elimination of Meis1 in adult cardiomyocytes triggers an increase in cardiomyocyte proliferation (Mahmoud et al., 2013). Research has revealed that Meis1 plays a crucial role in the transcriptional network governing cardiomyocyte cell cycle, hematopoietic stem cell maintenance, and cellular metabolism. These discoveries imply that Meis1 holds promise as a therapeutic target for a range of conditions, including modifying cancer metabolism, targeting cancer stem cells, expanding HSC populations, and promoting cardiac regeneration and possibly preventing cardiotoxicity (Aksoz et al., 2018; Meriç and Kocabas, 2022; Girgin and Kocabas, 2023).

Cardiotoxicity encompasses a spectrum of adverse effects on the heart due to drugs or other agents, which can lead to heart damage (Mercurio et al., 2016; Morelli et al., 2022), arrhythmias (Herrmann, 2020), and cardiomyopathy (Doser et al., 2009). In severe cases, it may result in heart failure (Bai et al., 2017; Sachinidis, 2020). This condition is particularly important in clinical medicine and drug development, notably in oncology, where some treatments have cardiotoxic effects, necessitating careful patient monitoring and potential treatment limitations (Cabeza et al., 2015; Henriksen, 2018; Meijers and de Boer, 2019; Khairnar et al., 2022). The downregulation of MEIS1 is associated with the enhanced maturation of oxidative phosphorylation during perinatal cardiomyocyte development, while Meis1 exerts inhibitory effects on angiotensin II-induced cardiomyocyte hypertrophy. Additionally, the restoration of Meis1 expression leads to improved electrophysiological function in cardiomyocytes (Zhang et al., 2016; Lindgren et al., 2019; Liu et al., 2022). Here we investigated MEIS1’s pivotal role in regulating cardiomyocyte cell cycle arrest as a promising therapeutic target. We aimed to provide a compelling pathway for enhancing cardiomyocyte renewal through MEIS1 inhibition. This is supported by investigations involving neonatal cardiomyocytes, wherein two novel small molecules, MEISi-1 and MEISi-2, are used to stimulate neonatal and adult cardiomyocyte proliferation and cytokinesis by downregulating MEIS target genes and cyclin-dependent kinase inhibitors (CDKIs). Additionally, the study included the effect of MEIS1 inhibition in early development via the cultivation and differentiation of human induced pluripotent stem cells (hiPSCs) into cardiomyocytes.

These findings could underscore the potential of MEIS inhibitors as a key regulator of cardiac gene expression, emphasizing their promise as therapeutic agents in regenerative cardiology.

## 2. Materials and methods

### 2.1 Isolation of rat neonatal ventricular cardiomyocytes

This protocol was conducted as previously described (Mahmoud et al., 2013). Hearts were extracted from 1- to 2-day-old rat pups after decapitation. To avoid contamination, the hearts were briefly immersed in ethanol within a sterile hood before being placed in an enzyme solution for digestion. Only the ventricular portion of the heart was dissected and it was placed in the enzyme solution. The enzyme solution consisted of 0.1% Pancreatin, and 50 mL of the solution was used for each batch. Subsequently, the hearts were incubated at 37 °C for 20 min with gentle agitation at 100–120 rpm, followed by centrifugation at 2000 rpm for 10 min. To remove fibroblast cells in the pellet, the isolated ventricular cardiomyocytes were seeded into a specialized cell culture medium (4 × 10 cm BD Falcon PRIMARIA tissue culture dish, cat# 353803) and incubated at 37 °C for 2 h. After the cells were gently collected, they were filtered through a 70–100 μm cell strainer and seeded in a cell culture medium precoated with gelatin, consisting of myocyte medium (3:1 DMEM: M199, Pen/Strep, L-Glutamine (2 mM), 10% normal rat serum, 5% FBS) at a density of 500,000 cells/mL and placed in a 37 °C incubator with 5% CO_2_.

### 2.2. Immunostaining in cardiomyocytes

This protocol was conducted as previously described (Mahmoud et al., 2013). Cardiomyocytes were expanded to a density of 50%–70% (5 × 10^5^ cells/6-well plates). They were treated with putative Meis1 inhibitors (0.1, 1, and 10 μM concentrations), and an increase in cardiomyocyte division rates was assessed by examining the levels of Ph3, AuroraB, and TnnT in the cells after 3–5 days. Immunostaining was conducted for this purpose. Following applications of putative Meis1 inhibitors, cardiomyocytes were fixed with 4% PFA (10 min at room temperature). Subsequently, the cells were permeabilized with 0.1% Triton X-100 for 15 min at room temperature. The cells were then blocked with 1% normal bovine serum (for 30 min) after washing. Next, the cells were incubated at room temperature with antibodies for phospho-histone H3 (PH3) (cell division marker, Ser10, 1:250 dilution, rabbit polyclonal, Millipore), Aurora B (cytokinesis marker, 1:250 dilution, rabbit polyclonal, Sigma), and cardiac troponin T (TnnT2) (cardiomyocyte marker, Thermo Scientific MS-295-P1, 1:200 dilution, mouse monoclonal). Detection was performed using secondary antibodies such as Alexa Fluor 488 donkey anti-mouse (Invitrogen, cat. no. A-21202, 1:400 dilution) and Alexa Fluor 555 donkey anti-rabbit (Invitrogen, 1:400 dilution), along with Hoechst 33342 (Invitrogen) DNA dye. The number of Ph3+TnnT2+ and AuroraB+TnnT2+ cardiomyocytes was determined using the GE Cytell imaging system.

### 2.3. Isolation of adult cardiomyocytes from mice heart

To isolate adult ventricular cardiac cells, we followed a previously described method (Mahmoud et al., 2013), starting with the treatment of adult cardiac tissue with collagenase. For the collection of pure and viable ventricular cardiomyocytes from adult mouse hearts, we utilized the EASYCELL-CM system from Harvard Apparatus. Following enzymatic perfusion of the heart, the cells were gathered and allowed to undergo gravity settlement. Within just 5 min, a pellet formed, which contained the cardiomyocytes. These cardiomyocytes were isolated and examined under a microscope.

### 2.4. Primary cardiac fibroblast isolation and culture from adult mouse heart

EASYCELL-CM and enzymatic processes allowed us to obtain both fibroblasts and cardiomyocytes. To separate the fibroblasts from the cardiomyocytes, we employed PRIMARI cell plates. The solution containing the fibroblasts was then seeded into two separate PRIMARI cell culture dishes (10 cm each), where the adhering fibroblasts were allowed to grow for 3–5 days in the cell culture.

### 2.5. Viability assay in primary cardiac fibroblasts

Cardiac fibroblasts were cultured in a 20% FBS DMEM medium and seeded in a 96-well plate with 5000 cells per well, allowing them to attach over 24 h. Subsequently, MEISi-1 and MEISi-2 compounds were introduced to the cells at about 5 μM for each well. The cells were then incubated at 37 °C for 72 h. Following the incubation period, MTS solution was introduced to the cell cultures and left to incubate for 2 h under standard culture conditions. Absorbance measurements were recorded at 490 nm using a microplate reader (Thermo Fisher Multimode Reader Varioskan Lux). To obtain accurate readings, the absorbance of the blank group was subtracted from the absorbance values of the samples.

### 2.6. IPSC culture and CM differentiation

hiPSCs (ATCC ACS-1026), generously provided by the Köse Lab at Yeditepe University, were cultured at 37 °C with regular medium changes. Cells were passaged every 3 to 4 days, maintaining 80%–90% confluence (Lian et al., 2013; Burridge et al., 2014; Karakikes et al., 2015). Passaging involved aspirating the medium, adding Versene, and transferring detached cells to Matrigel-coated plates at a 1:13 split ratio, with daily medium changes. Matrigel coating was prepared at 1.2 mg/mL in cold RPMI-1640, and the coated plates/flasks could be stored for up to 3 weeks. In the hiPSC-cardiomyocyte differentiation, we began with CDM3 + 6 μM CHIR on day 0, followed by CDM3 + 2 μM IWP2 on day 2, and continued with CDM3 medium until day 13 when cardiomyocytes were ready for expansion. Replating of differentiated hiPSC derived cardiomyocytes, suitable for cardiac expansion, occurred between days 10 and 14. This involved incubating cells with TrypLE Select, counting and replating them at a 1:10–20 split ratio in the desired culture system. Incubation was at 37 °C, 5% CO_2_, 21% O_2_, and 90% humidity, with gentle movements for even cell distribution.

### 2.7. Flow cytometric analysis in hiPSC-derived cardiomyocytes

To evaluate the quality and expansion rates of hiPSC-derived cardiomyocytes, the cells underwent a series of procedures. Initially, the cells were gathered, fixed, and subjected to immunocytochemical staining for the cardiac markers α-actinin and troponin-T. Subsequently, the cardiomyocytes were dissociated. Following cell counting, 100,000 cardiomyocytes were collected in 1.5-mL tubes and centrifuged at 200 × *g* for 3 min. The supernatant was then discarded, and 50 mL of 4% PFA solution was added to each tube for a 10-min incubation period. The cells were later resuspended at a concentration of 1 × 10^5^ cells in 50 mL of permeabilization buffer containing 5% BSA and 0.3% Triton X-100, and incubated for 30 min at 4 °C. Afterwards, the resuspended cardiomyocytes were placed in 50 mL of flow cytometry buffer, along with α-actinin antibody (1:300 dilution), troponin-T antibody (1:300 dilution), and a negative control containing 1 × 10^5^ cells in 50 mL of flow cytometry buffer. This mixture was incubated for 30 min at 4 °C. The cells underwent subsequent washing steps, including centrifugation at 200 × *g* for 5 min at 4 °C, with the supernatant being discarded and the washing process conducted twice. The final step involved resuspending the cells in 50 mL of flow cytometry buffer containing secondary antibodies (goat anti-mouse and goat anti-rabbit, both at 1:300 dilution) for analysis using a flow cytometer.

### 2.8. RT-PCR analysis of hiPSC differentiation into cardiomyocytes

Total RNA was prepared using the RNeasy Kit (Qiagen) and reverse-transcribed with the iScript cDNA Synthesis Kit (Bio-Rad) using random primers. qRT-PCR was performed using the i-Taq SBR Green Master Mix (Bio-Rad). The expression levels of the target genes were normalized to GAPDH levels (Table 1).

### 2.9. Cell viability assay in hiPSCs

hiPSCs were grown in m-TESR1 complete medium supplemented with 10 mM Y27632 and were seeded in a 96-well plate with 5000 cells per well and left for 24 h to attach. MEISi-1 was dissolved in dimethyl sulfoxide (DMSO) as a 100 mM stock solution. The molecule was then delivered to the cells at the various increased doses of MEISi-1 (10 nM up to 5 μM) for each well and incubated at 37 °C for 72 h. After incubation, MTS solution was added to the cells and incubated for 2 h under standard culture conditions. Absorbance was measured at 490 nm using a microplate reader (Multimode Reader Varioskan Lux, Thermo Fisher). The absorbance of the blank group was subtracted from the absorbance of the samples.

### 2.10. Long-term and short-term MEISi-1 treatment of hiPSCs

The hiPSCs were thawed and seeded onto Matrigel-coated six-well plates at a cell density of 0.8 × 10^4^ cells/cm^2^ in mTeSR1 medium for 4 days. Afterward, the hiPSCs were treated with 5 μM MEIS inhibitor (MEISi) for 18 days, with medium changes every 2 days. From day 10 onwards, the experiment was continued without further medium changes for an additional 8 days. On day 18, the cells were collected for qPCR experiments.

In the short-term MEISi treatment method, hiPSCs are initially cultured on Matrigel-coated 6-well plates for 4 days using mTesR medium with the same density of cells. Subsequently, to induce mesoderm progenitor cell formation, a 6 μM GSK3 inhibitor (CHIR) is introduced in a differentiation medium containing RPMI, ascorbic acid, and albumin, and the cells are left to incubate for 48 h. After this stage, the differentiation medium (CMD3) is prepared, supplemented with WNT inhibitor (IWP2) and MEIS inhibitor1, and the cells are cultured for an additional 3 days to promote the differentiation of cardiac mesoderm and cardiac progenitor cells. Throughout the process, cellular changes are monitored with a qPCR experiment to investigate whether MEIS inhibitors play a role similar to WNT inhibitors in guiding mesoderm cells towards a cardiac mesoderm or cardiac progenitor cell fate.

### 2.11. Analysis of cardiac tissue post-MEIS injections

To assess the expression of ventricular cardiomyocyte cell cycle regulators in cardiac tissue, we employed real-time qPCR following MEISi-1 and MEISi-2 injections into the animals, as outlined in our previous study (Turan and Kocabas, 2020). Whole cardiac tissue samples were collected and half of them subsequently powdered in liquid nitrogen using a mortar. Half of the cardiac tissue was used for parafilm sectioning and TnnT/Ph3 immunohistochemistry studies as outlined previously ((Mahmoud et al., 2013). RNA isolation was performed using the TRIzol method. RNA concentration was determined with a NanoDrop (Thermo Fisher). For each tissue sample, 5 μg of RNA was converted into cDNA using random primers and the ProtoScript II First Strand cDNA Synthesis Kit (NEB, Cat. No: E656). Subsequently, the samples were stored at −20 °C after dilution. Gene-specific primers (Table 2) were selected using NIH primer depot (http://mouseprimerdepot.nci.nih.gov) and ordered from Sentebiolab in Türkiye. The desired gene regions were then amplified from the cDNAs using a Bio-Rad FX96 Touch Real-Time qPCR Detection System, following the cycling conditions of 95 °C for 10 min, 95 °C for 10 s, 60 °C for 20 s, and 72 °C for 30 s (30 cycles). The expression of each amplified potential modulator gene was normalized against the GAPDH content using the ΔΔCt method.

### 2.12. Anesthesia

The mice were anesthetized as previously reported (Golan-Lagziel et al., 2018). In brief, they were placed in an induction chamber (Hugo Sachs Electronik) and 2%–3% vol/vol isoflurane delivered by 95% oxygen (1 L/min) was administered. After establishing narcosis, the mice were transferred to a surgical platform (Kent Scientific), equipped with a temperature control module (Kent Scientific), and fixed in a supine position with a rectal probe inserted for temperature maintenance at 37 °C throughout the entire procedure. The isoflurane concentration was regulated at 1.5% vol/vol using a vaporizer (Hugo Sachs Electronik) to maintain narcosis. Fentanyl (0.05 mg/kg body weight) was injected intraperitoneally for analgesia in the beginning of the experiment; additional doses (0.025 mg/kg) were given every 45 min. Depth of anesthesia was evaluated by the toe pinch reflex. Subsequently, once a negative response in the toe pinch reflex was confirmed, we could initiate the following experiments.

### 2.13. Echocardiography

Echocardiographic studies were conducted using a VEVO-2100 Imaging ultrasonographic system (VisualSonic, Toronto, Canada) at a resolution of 100 dpi. After anesthesia induction, a 12.5 MHz transducer was applied to the left hemithorax and two-dimensional M-mode images from the short-axis view were acquired. LV end-diastolic and end-systolic diameters, as well as LV anterior and posterior wall thicknesses, were measured using the leading-edge convention of the American Society of Echocardiography. The LV ejection fraction (EF) percentage was computed as EF (%) = (LVIDd3-LVIDs3) / LVIDd3 × 100, where LVIDd3 and LVIDs3 represent LV end-diastolic and end-systolic diameters, respectively.

### 2.14. ECG

Following anesthesia induction, three subcutaneous needle electrodes (29 G, AD Instruments) were inserted in both the upper and left lower limbs of the mice to allow recording of a Lead I ECG. To record and analyze ECGs, an amplifier (AD Instruments), a PowerLab system (AD Instruments), and LabChart Pro software (AD Instruments) were used. The ECG data were derived from 5-min recordings.

### 2.15. Statistical analysis

The data are presented as the mean ± standard error of the mean (SEM). Significance levels were determined using a two-tailed Student’s t-test and one-way ANOVA. Statistical significance in the echocardiography data was evaluated using the two-sided Mann–Whitney test. Statistical significance was ascribed to results with p-values less than 0.05.

### 2.16. Approvals

All animal and human studies are approved by the local institutions of Yeditepe University, İstanbul, Türkiye (Ethical Committee Approval #673) and LMU Munich, Munich, Germany.

## 3. Results

### 3.1. MEIS1 inhibitors stimulate cardiomyocyte proliferation and cytokinesis

**Figure 1:**
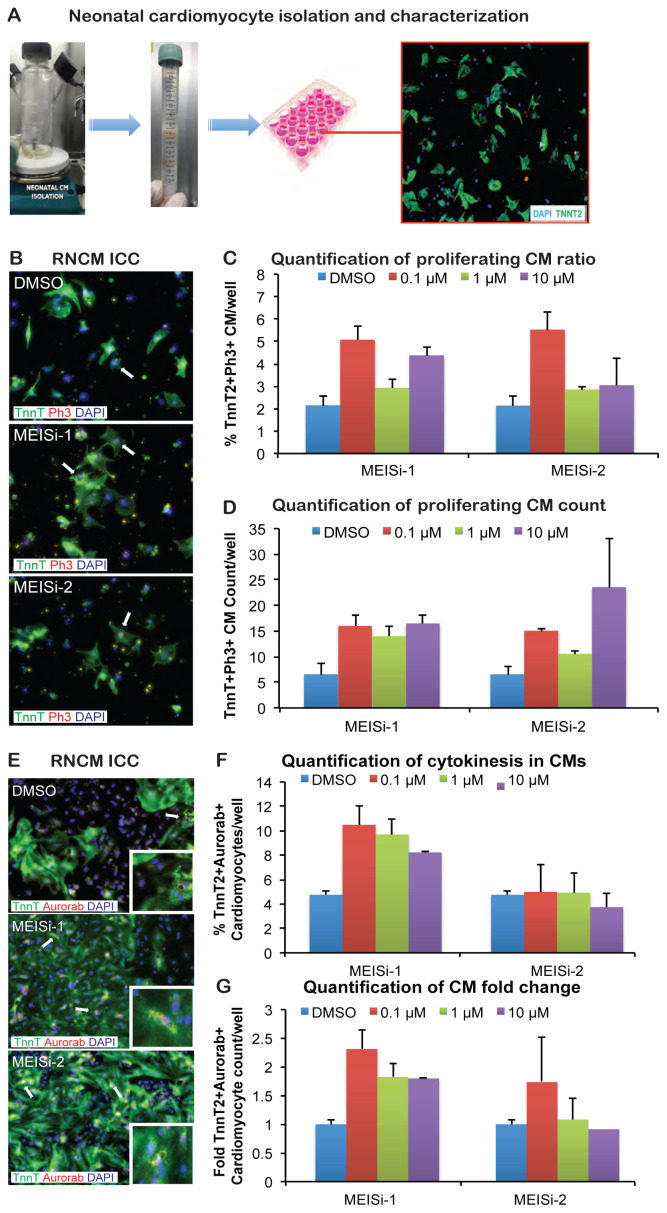
Treatment of neonatal cardiomyocytes with MEIS inhibitors. A) Schematic of rat neonatal cardiac tissue digestion and isolation. B) Immunostaining of rat neonatal ventricular cardiomyocytes (RNCMs) with Ph3 (red), TnnT2 (green), and DAPI (blue) after treatment with DMSO (control), MEISi-1, and MEISi-2. C) Quantification of proliferating ventricular cardiomyocytes (% of TnnT2+Ph3+ CMs/well) with different doses of MEISi-1 and MEISi-2. D) Quantification of proliferating ventricular cardiomyocytes counts (number of TnnT2+Ph3+ CMs/well) with different doses of MEISi-1 and MEISi-2. E) Immunostaining of RNCMs with AuroraB (red), TnnT2 (green), and DAPI (blue) after treatment with DMSO (control), MEISi-1, and MEISi-2. F) Quantification of cytokinesis in ventricular cardiomyocytes (% of TnnT2+AuroraB+ CMs/well) with different doses of MEISi-1 and MEISi-2. G) Quantification of cytokinesis in ventricular cardiomyocytes count (fold difference of TnnT2+AuroraB+ CMs/well compared to DMSO) with different doses of MEISi-1 and MEISi-2. n = 3.

To investigate the dynamics of cell division within cardiomyocytes, we employed rat neonatal cardiomyocytes (RNCMs) as a model system (Figure 1A). Then cellular division events were meticulously examined using immunostaining techniques. Upon successful isolation of RNCMs, they were cultured in 96-well plates and subjected to a progressive gradient of MEIS1 inhibitors at concentrations of 0.1, 1, and 10 μM. Following a 3-day incubation period, the cardiomyocytes were fixed and subsequently subjected to immunostaining using antibodies targeting specific markers: TnnT2 for cardiomyocytes, Ph3 for proliferation, and AuroraB for cytokinesis.

The research analysis was primarily centered on quantifying actively dividing cardiomyocytes, defined as TnnT2+Ph3+. In addition, an evaluation of cytokinesis rates was conducted by examining TnnT2+AuroraB+ markers (Figure 1B). Our findings revealed a substantial augmentation in both the proportion (Figure 1C) and number of TnnT2+Ph3+ cardiomyocytes (Figure 1D), demonstrating an increase of up to 2.5 times. Moreover, a similar enhancement, amounting to a twofold increase, is clearly observed in the population of cardiomyocytes undergoing cytokinesis subsequent to MEIS1 inhibitor treatment, as visually represented in Figures 1E–1G.

In conclusion, our study using neonatal rat cardiomyocytes and MEIS1 inhibitors demonstrated a substantial increase in actively dividing cardiomyocytes and those undergoing cytokinesis. These findings shed light on the potential for regulating cardiomyocyte division and offer valuable insights into the potential mechanisms that could be harnessed for cardiac regeneration and repair in the adult heart.

### 3.2. MEIS1 inhibitors promote adult ventricular cardiomyocyte proliferation and may inhibit fibroblast growth

**Figure 2:**
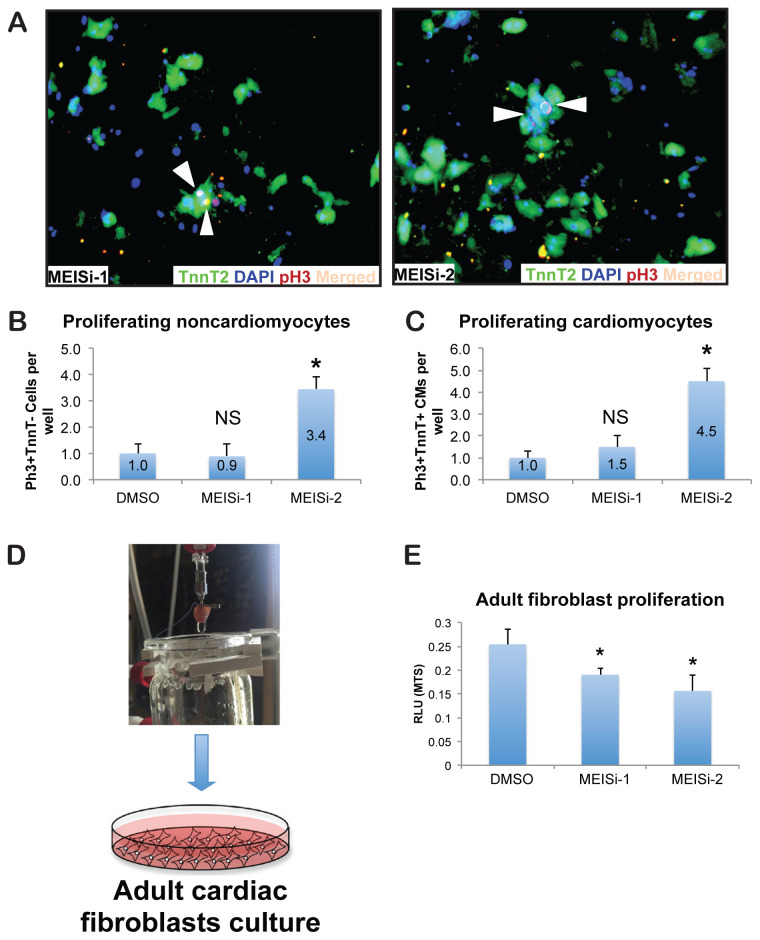
Treatment of mature ventricular cardiomyocytes and noncardiomyocytes with MEIS1 inhibitors. A) Immunostaining of adult mouse ventricular cardiomyocytes (CMs) with Ph3 (red), TnnT2 (green), and DAPI (blue) after treatments with MEISi-1 and MEISi-2. B) Quantification of proliferating noncardiomyocytes (% of TnnT2-Ph3+ CMs/well) and C) Quantification of proliferating ventricular cardiomyocytes (% of TnnT2+Ph3+ CMs/well) after MEISi-1 and MEISi-2 treatments in comparison to DMSO. D) Schematic of isolation and culture of cardiac fibroblasts. E) Viability analysis of cardiac fibroblasts after treatments with MEISi-1 and MEISi-2. n = 3, *p < 0.05, NS: Not significant.

Cardiomyocytes in the adult heart often enter a quiescent state, displaying limited participation in the active cell cycle. To explore the dynamics of cell division within adult cardiac tissue, adult ventricular cardiomyocytes as well as noncardiomyocytes were isolated and cultured, and MEIS1 inhibitors at concentrations of 5 μM were applied. After a 3-day incubation period, immunostaining was performed using TnnT2 for cardiomyocytes and Ph3 for proliferation (Figure 2A).

Our findings demonstrate a significant increase in the number of noncardiomyocytes (TnnT2-Ph3+) (Figure 2B) and cardiomyocytes (TnnT2+Ph3+) (Figure 2C) post-MEISi-2 treatments only. MEISi-1 did not show any effect on the proliferation of primary ventricular cells (Figures 2B, 2C). Next, we assessed the effect of MEIS1 inhibitors in cultured cardiac fibroblasts (Figure 2D). Intriguingly, both MEISi-1 and MEISi-2 treatments reduced cardiac fibroblast proliferation in vitro (Figure 2E).

In conclusion, our research conducted with adult cardiac tissue and the application of MEIS1 inhibitors underscores a significant augmentation in actively dividing ventricular cardiomyocytes and suggests the potential inhibition of fibroblasts originating from adult tissue.

### 3.3. Expression of Meis1/2 increased after cardiac differentiation of human IPSCs

**Figure 3:**
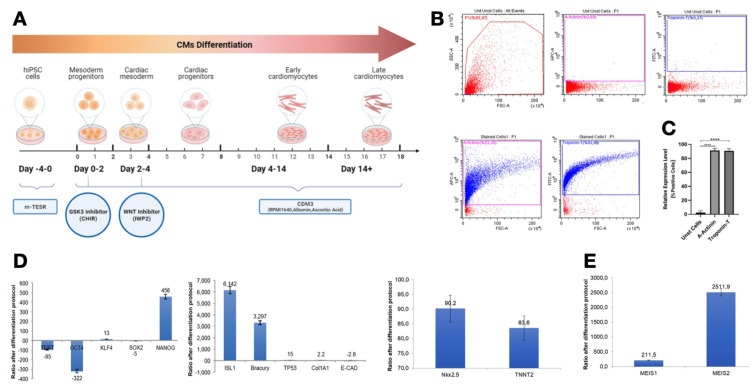
hiPSC to cardiomyocyte differentiation and Meis1/2 expression analysis. A) Schematic of iPSC to cardiomyocyte differentiation procedure. B) Representative graphs of flow cytometry analysis of cardiomyocyte differentiation. C) Quantification of TnnT and actinin in human iPSC derived cardiomyocytes (hiPSC-CMs). D) Analysis of gene expression of pluripotency, cardiac mesodermal, and cardiac markers after cardiomyocyte differentiation of iPSC. E) Analysis of Meis1 and Meis2 expression after cardiomyocyte differentiation of iPSC. Unst: Unstained, n = 3, *p < 0.05, **p < 0.001.

Next we wanted to assess if MEIS expression plays a role in cardiac differentiation in hiPSCs. To induce cardiac differentiation in these cells, we employed a strategic approach utilizing GSK3 and WNT inhibitors, as delineated in Figure 3A. These cells were cultured on Matrigel-coated six-well plates and maintained in mTeSR1 medium for 4 days. The differentiation process commenced with the introduction of CDM3 medium and CHIR99021 over 48 h, leading to the emergence of Brachyury-expressing cells. Subsequently, we introduced the IWP2 inhibitor for an additional 48 h to guide the cells towards a cardiac fate. The culture medium was refreshed every 2 days, and functional contracting cardiomyocytes were observed after 13 days (Figure 3B). This was evident with the proportions of TnnT+ and actinin+ cells in hiPSC derived cardiomyocytes (Figure 3C).

We conducted an in-depth examination of gene expression patterns through real-time reverse-transcription PCR (RT-PCR). Our findings were consistent with established differentiation strategies and embryonic development principles. We observed a rapid downregulation of pluripotency markers OCT4, TERT, and Sox2, concomitant with the upregulation of cardiac mesoderm markers ISL1 and Brachyury (Figure 3D). Notably, both early and late cardiomyocyte markers Nkx2.5 and TNNT2 exhibited robust expression, as depicted in Figure 3D. Furthermore, it is noteworthy that Meis1 and Meis2 displayed significant upregulation (Figure 3E).

In summary, the findings suggest that the upregulation of Meis1 and Meis2 genes following cardiac differentiation of hiPSCs underscores their significant roles in cardiac development.

### 3.4. Effect of long-term and short-term MEIS1 inhibition in hiPSC differentiation

**Figure 4:**
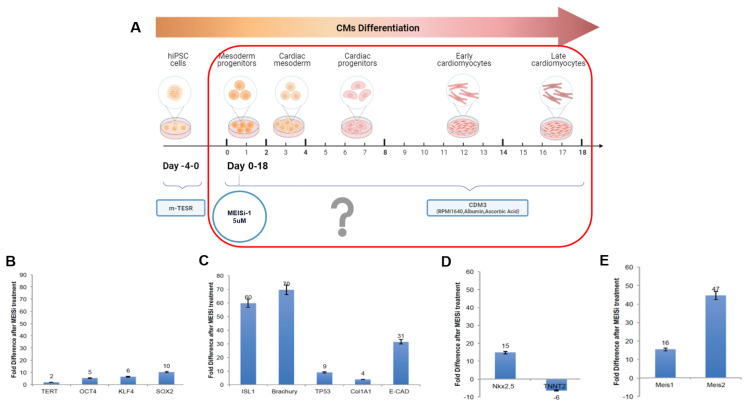
Effect of long-term MEIS1 inhibition in iPSC differentiation. A) Schematic of long-term MEIS1 inhibition in iPSCs. Analysis of gene expression of B) pluripotency, C) cardiac mesodermal, and D) cardiac markers. E) Analysis of Meis1 and Meis2 expression during cardiomyocyte differentiation of iPSC in comparison to no treatment and long-term MEIS1 inhibition. n = 3.

We initially assessed whether inhibiting the MEIS pathway resulted in any cytotoxicity for iPSCs. To this end, we assessed hiPSCs’ cell viability and mitochondrial activity post-MEISi-1 treatments using the MTS tetrazolium assay. These experiments, which included a range of MEISi-1 doses, did not show significant changes in mitochondrial activity or cell viability compared to the control group treated with DMSO, the solvent for the MEIS1 inhibitor (Figure S1). Then the MEIS1 inhibitor treatment protocol was designed to explore the impact of MEISi-1 on hiPSCs and monitor gene expression alterations throughout the experiment (Figure 4A). The hiPSCs were initially grown on Matrigel-coated plates and then subjected to MEISi-1 for an extended duration, with routine medium changes up to day 10. Following this, the medium remained unchanged for the subsequent 8 days. Cells collected on day 18 were employed for qPCR analysis, facilitating the assessment of gene expression profiles influenced by MEIS1i treatment.

We observed that MEIS1 inhibition results in the upregulation of pluripotency markers, including TERT, OCT4, and Sox2 expression (Figure 4B) following MEISi-1 treatments in comparison to the control (DMSO). Additionally, cardiac mesoderm markers ISL1 and Brachyury exhibited increased expression, indicating increased mesodermal differentiation after MEISi-1 treatments (Figure 4C). Conversely, later cardiomyocyte marker TNNT2 showed downregulation post-MEIS1 inhibition, suggesting a hindrance in terminal cardiomyocyte differentiation (Figure 4D). Intriguingly, Meis1 and Meis2 exhibited a modest upregulation (16- and 47-fold increases, respectively) (Figure 4E), which in turn is a significant downregulation compared to no treatment as seen in the previous study (Meis1: 211- and Meis2: 2511-fold increase) (Figure 3E).

**Figure 5:**
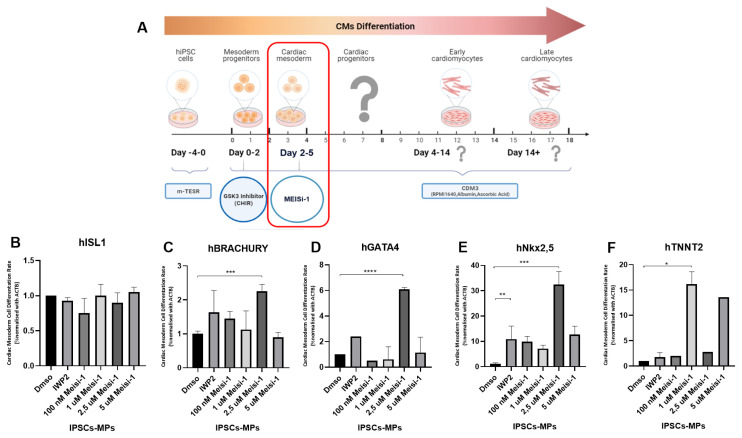
Effect of short-term MEIS1 inhibition in IPSC differentiation. A) Schematic of short-term MEIS1 inhibition in iPSCs. Analysis of B) ISL1, C) Brachyury, and D) GATA4 gene expression after short-term MEIS1 inhibition. Cardiac mesodermal cell differentiation rates refer to gene expression levels normalized to ACTB gene expression. Analysis of cardiac marker E) Nkx2.5 and F) Tnnt2 expression after short-term MEIS1 inhibition. n = 3, *p < 0.05, **p < 0.001.

In the short-term MEISi-1 treatment method, hiPSCs are initially cultured for 4 days. Subsequently, a 6 μM GSK3 inhibitor (CHIR) is introduced into a differentiation medium and the cells are incubated for 48 h to induce mesoderm progenitor cell formation (Figure 5A). After this stage, the differentiation medium (CMD3), supplemented with WNT inhibitor (IWP2) and MEIS inhibitor 1 separately, is used for an additional 3-day culture to promote the differentiation from mesoderm stage to cardiac mesoderm (Figure S2). We analyzed the expression of ISL1 (Figure 5B), Brachyury (Figure 5C), and GATA4 (Figure 5D) after short-term MEIS inhibition. Interestingly, consistent findings for ISL1 gene expression were observed after short-term MEIS1 inhibition, indicating no significant changes. Another mesoderm marker, Brachyury expression, showed upregulation (Figure 5C), highlighting the enhanced mesodermal differentiation under MEIS1 inhibition conditions. Furthermore, GATA4 (Figure 5D) expression was also notably upregulated, indicating that MEIS1 inhibition plays a pivotal role in promoting mesoderm formation and subsequent cardiac mesoderm commitment. These results collectively demonstrate the positive regulatory effect of MEIS1 inhibition on key cardiac mesoderm markers, underscoring its potential in influencing cardiac differentiation processes.

Moreover, analysis of early (Nkx2.5) (Figure 5E) and late (Tnnt2) (Figure 5F) cardiac markers expression after short-term MEIS1 inhibition showed notable upregulation in their expression levels. This indicates that the MEIS1 inhibition during the differentiation process had a stimulatory effect on the activation of these cardiac markers. The increased expression of Nkx2.5 and Tnnt2 suggests that MEIS1 inhibition enhances the proper differentiation of cardiac mesoderm progenitor cells and their progression into more mature cardiac cell lineages. These findings highlight the regulatory role of MEIS1 in cardiac development and the potential for MEIS1 inhibitors to positively influence cardiac lineage commitment and maturation.

The analysis of various markers, including ISL1, Brachyury, GATA4, Nkx2.5, and Tnnt2, offered insights into the effects of long- and short-term MEIS1 inhibition on hiPSC differentiation. Overall, our findings shed light on the intricate interplay between MEIS1 inhibition and the regulation of pluripotency and cardiac differentiation in iPSCs, providing valuable insights for future research in this field.

### 3.5. Investigating the effect of MEIS1 inhibitors on ventricular cardiomyocyte proliferation and gene expression

**Figure 6:**
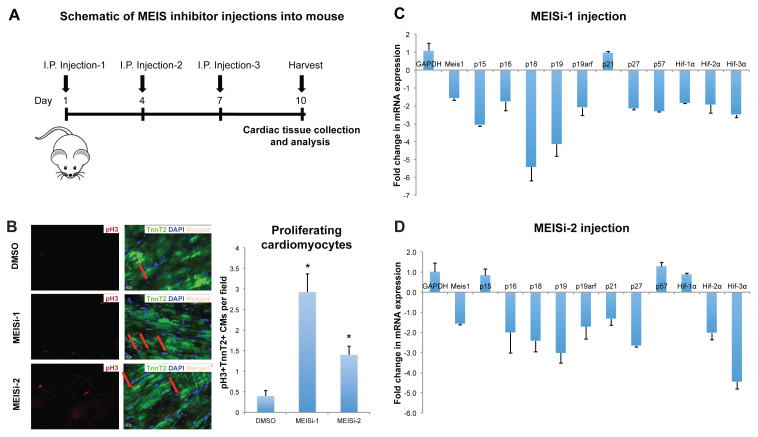
Analysis of cardiac tissue post-MEIS1 inhibition in vivo. A) Schematic of MEIS1 inhibitor injection into mice. B) Immunostaining (left) and quantification (right) of left ventricle of mouse heart with Ph3 (red), TnnT2 (green), and DAPI (blue) after injections with DMSO (control), MEISi-1, and MEISi-2. Analysis of gene expression cardiac tissue of C) MEISi1 and D) MEISi-2 injected mice. n = 3, *p < 0.05.

These findings suggest that MEIS1 inhibitors may trigger cell cycle activation in ventricular cardiomyocytes, especially due to the decrease in the expression of CDKIs, which negatively regulate the cell cycle. To investigate this, we analyzed cardiac tissue after injection of MEIS1 inhibitors into mice (Figure 6A). Studies involved mouse heart tissues to investigate the live imaging effects of Meis1 inhibitors on cardiomyocyte proliferation via immunohistochemistry (Figure 6B) and expression of CDKIs after serial MEISi-1 and MEISi-2 injections by qPCR (Figure 6C).

Immunostaining and quantitative analyses conducted in the left ventricle of mouse hearts following injections of DMSO (control), MEISi-1, and MEISi-2 distinctly demonstrate that MEIS1 inhibitors significantly enhance the proliferation of ventricular cardiomyocytes (Figure 6B). Within the left ventricular regions, a remarkable increase in the number of cells labeled with TnnT2+Ph3+ (a proliferation marker) is observed. These findings indicate that MEIS1 inhibitors stimulate the division of ventricular cells, suggesting this process as a promising target for cardiovascular regeneration.

Previous studies demonstrated that the expression of CDKIs decreases in Meis1 knockout experiments, and these inhibitors directly regulate the expression of genes such as p21, Hif-1α, and Hif-2α. Here we examined whether the MEIS1 inhibitors we developed trigger similar gene regulation in cardiac tissue. Following MEISi applications, we observed a reduction in the expression of genes targeted by Meis1, including Hif-2α, and several CDKIs including p16, p18, p19, p19arf, and p27 after both MEISi-1 (Figure 6C) and MEISi-2 injections (Figure 6D). The efficacy of the Meis1 inhibitors MEISi-1 and MEISi-2 became apparent through the observed reduction in Meis1 as well as mRNA expression of the genes targeted by Meis1.

In mouse heart tissue, we observed an increase in ventricular cardiomyocyte proliferation following MEIS1 inhibitor injections. These findings indicate that MEIS1 inhibitors have the potential to activate ventricular cardiomyocyte cell cycles by downregulating CDKIs. The reduced expression of CDKIs and Meis1-targeted genes further supports the effectiveness of these MEIS1 inhibitors, suggesting their promise for promoting cardiac cell division and cardiovascular regeneration.

### 3.6. Evaluating the short-term effect of MEIS1 inhibition on cardiac structure and function in vivo

Given the above results implying a potential role for MEIS1 inhibition in activating cell proliferation in cardiomyocytes, we assessed the impact of MEIS1 inhibition on the heart in vivo. Compared to control mice, MEIS1 inhibitor-treated animals showed a trend towards an increased heart weight and heart weight/tibia length ratio (Figures S3A–S3C).

To investigate the effects of MEIS1 inhibitor treatment on cardiac dimensions and ejection fraction, echocardiography was performed. LV diameter and ejection fraction, however, were not affected by MEIS1 inhibition (Figures S3D, S3E). In line with the gross anatomy findings, a nonsignificant trend towards an increased diastolic thickness of the interventricular septum (Figures S3F, S3G) was observed.

To evaluate the potential effects of MEIS1 inhibition on cardiac conduction, ECGs were analyzed (Figures S3H–S3M). None of the ECG parameters including heart rate, P wave duration, PR interval, QRS duration, or QTc interval was significantly altered after MEIS1 inhibition.

## 4. Discussion

Heart failure is a pervasive medical condition, affecting millions of individuals worldwide. At the heart of its pathophysiology lies the heart’s reduced contractile force, stemming from the replacement of deceased cardiomyocytes, following cardiac events such as ischemia, with noncontractile fibrotic tissue. Although the general mammalian heart is considered incapable of regeneration, a limited number of myocytes do exhibit cell cycling. However, this phenomenon falls short of achieving substantial functional recovery postheart attack. While the mechanisms behind cardiomyocyte cell cycling in adult mammalian hearts remain incompletely understood, the activation of cardiomyocytes is considered a promising strategy for cardiac regeneration.

Research on the development of small-molecule compounds has primarily focused on revealing substances that could facilitate the transformation of various stem cells or progenitors into cardiac cells, rather than concentrating on regulators of the cardiomyocyte cell cycle (Choi et al., 2013). Notable among the small molecules discovered for this purpose are those such as SB-203580 (Perea-Gil et al., 2022), CHIR99021 (Hesselbarth et al., 2021), ERK (Ba et al., 2019; Kubin et al., 2020), and CamKII inhibitors (Hegyi et al., 2019; Helmstadter et al., 2021; Zhang et al., 2022), which target stem cells and progenitors. These compounds exert their effects on signaling mechanisms like MAPK (Becatti et al., 2012; Chen et al., 2023) and GSK-3β (Wang et al., 2022a, 2022b). Additionally, there is knowledge that small molecules like NBI-31772 (Kim et al., 2016) and bromoindirubin-30-oxime (BIO) (Guo et al., 2020) have a proliferative effect in zebrafish and mammalian heart muscle. However, it is important to note that, thus far, these investigations have not yielded outcomes at the desired level in terms of heart regeneration. This limitation can be largely attributed to the absence of comprehensive tools for studying modulators of mammalian heart regeneration.

The identification of novel cardiogenic factors, such as Meis1, has provided an innovative foundation for the development of treatments targeting the cardiomyocyte cell cycle. Our previous molecular studies have revealed that Meis1 exerts transcriptional influence, turning factors that typically exert a negative impact on the cell cycle, like the p21 and INK4b loci, into positive regulators and negatively affecting cell division in cardiomyocytes (Mahmoud et al., 2013). Interestingly, these two gene families are capable of inhibiting the cell cycle at different stages, underscoring the role of cell cycle arrest as the fundamental mechanism in both cardiac cell renewal and the prevention of excessive proliferation in cardiomyocytes.

The majority of studies suggest that in adult cardiomyocytes the levels of CDKIs increase and their activities are associated with positive cell cycle regulators such as cyclins and CDKs (Brooks et al., 1997, 1998; Poolman and Brooks, 1998; Poolman et al., 1998). Several studies have demonstrated that Cdk2 and c-myc serve as cell cycle regulators, contributing to the upregulation of CDKs like cyclin CDK4 and CDK4, thereby promoting cardiomyocyte growth (Perez-Roger et al., 1999). Moreover, the deletion of CDKIs p27Kip1 or p21Cip1 in cardiomyocytes leads to S-phase progression, consequently enhancing cardiomyocyte proliferation and increasing heart size (Poolman et al., 1998, 1999). Recent research has shown that Meis1 deletion induces the upregulation of CDKs and downregulation of CDKIs like p16, p15, p19ARF, p21, and p57 (Kocabas et al., 2012; Mahmoud et al., 2013). Additionally, Meis1 deletion results in increased downregulation of positive cell cycle regulators such as MCM3, Chek1, and Ccnd2, and an upregulation of negative cell cycle regulators like APbb1, TP53, and Gpr132 (Kocabas et al., 2012; Mahmoud et al., 2013). Targeting Meis1 represents a viable mechanism for inducing cardiomyocyte proliferation.

Research into small molecules that stimulate cardiomyocyte regeneration is advancing rapidly. Our study offers significant insights into the effects of MEIS inhibitors on cardiomyocyte proliferation and gene expression. In neonatal rat cardiomyocytes, MEIS inhibitors significantly increased the number of actively dividing and cytokinesis cells, suggesting their potential for cardiac regeneration. Similarly, in adult cardiac tissue, these inhibitors reactivated the cell cycle, enhancing cardiomyocyte proliferation, and reduced cardiac fibroblast proliferation, impacting noncardiomyocyte cells within the heart. Additionally, investigations with hiPSCs undergoing cardiac differentiation revealed upregulation of MEIS1 and MEIS2, underscoring their roles in cardiac development (Sun et al., 2019). Recent research further highlights MEIS2’s role in calcific aortic valve disease (CAVD), where its inhibition promotes osteoblastic transdifferentiation and reduces Notch1 and Twist1 expression, making MEIS2 a potential target for CAVD prevention.

A recent study reexamined ISL1’s role in human embryonic stem cell-based cardiac development, revealing that ISL1 accelerates cardiomyocyte differentiation rather than stabilizing precursor cells (Quaranta et al., 2018). Depletion of ISL1 delays cardiac differentiation and alters cardiomyocyte identity, as ISL1 interacts with retinoic acid signaling and MEIS2, competing with the retinoic acid pathway and the atrial specifier NR2F1 for cardiomyocyte fate. These findings provide valuable insights for cardiac regeneration strategies. Although the neonatal heart has inherent regenerative potential through cardiomyocyte proliferation, this capacity diminishes after postnatal day 7. We previously demonstrated that deleting MEIS1 in mouse cardiomyocytes extends the postnatal proliferative period and reactivates cardiomyocyte mitotic activity in adult hearts. MEIS1 plays a crucial role in activating critical CDK inhibitors, such as p15, p16, and p21, marking it as a fundamental regulator of cardiomyocyte proliferation and a promising therapeutic target for heart regeneration (Mahmoud et al., 2013). Our observations with mouse heart tissue further support the potential of MEIS1 inhibitors to stimulate ventricular cardiomyocyte proliferation, downregulate CDKIs, and enhance cardiovascular regeneration, highlighting their prospective role in developing regenerative treatment strategies.

A recent study investigated the role of Meis1 in ischemic arrhythmias in mice (Lian et al., 2013). Meis1 overexpression was found to reduce ventricular arrhythmias and improve cardiac conduction velocity, partly by restoring the function of cardiac Na^+^ channels. Additionally, the study revealed that E3 ubiquitin ligase CDC20 plays a role in this process, highlighting a new mechanism for Na_V_1.5 channel dysregulation in infarcted hearts. Fascinatingly, through the utilization of a Cre-dependent CasRx knock-in mouse model enabling precise gene suppression, another research team effectively downregulated Meis1 and Hoxb13 expression in ventricular cardiomyocytes (Li et al., 2022). This intervention spurred cardiac regeneration after a myocardial infarction while also inhibiting the lncRNA Mhrt.

MEIS proteins have a well-established connection with cancer, being implicated in tumorigenesis, metastasis, and invasion. In different cellular contexts, they can act as either tumor suppressors or oncogenes, and their expression frequently becomes dysregulated in various cancers (Girgin et al., 2020). Studies have indicated their upregulation in cancers such as leukemia, lymphoma, thymoma, pancreas, glioma, and glioblastoma, as well as downregulation in cervical, uterine, rectal, and colon cancers. It is noteworthy that, within each cancer type, at least one subtype exhibits elevated MEIS expression. Furthermore, research has identified the potential of MEIS proteins and their associated factors as diagnostic or therapeutic biomarkers for various diseases.

There is a historical association between Meis1 and acute leukemia, a challenging hematological disease characterized by resistance to therapy and frequent relapses. Meis1 has been implicated in the pathogenesis of various cancers, with historical observations linking its overexpression to both acute lymphoblastic leukemia and acute myeloid leukemia (Meriç and Kocabas, 2022). Elevated MEIS1 expression in leukemic blast samples is associated with resistance to conventional treatments. A recent study indicated that MEIS inhibitors have the potential to reduce the viability of leukemia stem cells by inducing apoptosis, suggesting their possible use in limiting leukemia relapse and overcoming chemotherapeutic resistance (Mercurio et al., 2016). These findings imply that MEIS inhibitors could be promising for the treatment of leukemia and potentially other disorders characterized by similar resistance mechanisms.

In addition, we have recently shown that newly developed MEIS inhibitors selectively hinder the growth of prostate cancer cells with high MEIS expression, triggering apoptosis (Girgin and Kocabas, 2023). MEIS inhibition effectively reduced the viability of various prostate cancer cell lines and increased apoptosis, particularly in cells with elevated MEIS levels. These findings suggested the potential use of MEIS inhibitors for targeting high MEIS-expressing prostate cancer, although further research is needed before clinical application. The observations in leukemia and solid cancer cases indicate the potential utility of MEIS inhibitors in the management of MEIS-dependent or MEIS-positive malignancies.

It is intriguing that our findings reveal differential effects of MEIS inhibition on cardiac fibroblasts compared to cardiomyocytes. To understand why MEIS1 impacts the cell cycle of these cell types differently, it is crucial to recognize that the precise mechanisms by which MEIS1 and its cofactors regulate cardiomyocytes versus fibroblasts are not yet fully elucidated. MEIS1 plays a role in regulating gene expression programs specific to each cell type, but the exact mechanisms by which MEIS1 and its cofactors differentially influence cardiac fibroblasts and cardiomyocytes remain unclear. In fibroblasts, MEIS1 is part of a regulatory network that maintains fibroblast-specific gene expression and suppresses cardiomyocyte-specific genes (Rastegar-Pouyani et al., 2017; Golan-Lagziel et al., 2018; Alam et al., 2019). This function is crucial for preserving fibroblast identity and function. However, the impact of MEIS1 on the cell cycle and gene expression in cardiomyocytes is less well defined and may involve interactions with other transcription factors and regulatory elements that are unique to cardiomyocytes. Our current understanding is limited regarding how MEIS1 and its cofactors are expressed and function differently in cardiac fibroblasts versus cardiomyocytes. This gap in knowledge suggests that MEIS1’s role in these cell types could be more complex than previously thought, potentially involving distinct regulatory networks and mechanisms that influence cell cycle dynamics and differentiation. Further research is needed to elucidate these differences and understand how MEIS1’s actions in these cell types contribute to their unique behaviors and responses.

Heart failure, a widespread medical condition marked by reduced cardiac contractility and limited cardiomyocyte regeneration, underscores the need for innovative strategies in cardiac regeneration. While the mammalian heart exhibits regenerative potential in neonatal stages, this capacity diminishes after postnatal day 7. Studies have identified Meis1 as a crucial regulator of the cardiomyocyte cell cycle, offering promise in enhancing cardiac regeneration. The use of MEIS1 inhibitors, which promote cardiomyocyte proliferation and gene modulation, may serve as a potential therapeutic approach to stimulate cardiac repair. In addition to their relevance in cardiac regeneration, MEIS inhibitors hold promise in the context of cancer. MEIS proteins are associated with tumorigenesis and research indicates that MEIS inhibitors have the potential to reduce the viability of leukemia stem cells and induce apoptosis, suggesting their utility in addressing chemotherapeutic resistance in leukemia. Moreover, newly developed MEIS inhibitors exhibit efficacy in selectively blocking prostate cancer cells with high MEIS expression, triggering apoptosis. These findings highlight the multifaceted potential of MEIS inhibitors in addressing cardiomyocyte regeneration and managing MEIS-dependent or MEIS-positive malignancies.

## Supplementary Materials

Figure S1 (related to Figure 3) Analysis of iPSC viability and mitochondrial activity via MTS tetrazolium assay post-MEISi-1 treatments. Increasing doses of MEISi-1 (10 nM up to 5 μM) were assessed in hiPSCs for cell viability.

Figure S2 (related to Figure 5) Effect of short-term MEIS1 inhibition in TBXT expression during IPSC differentiation. n = 3, ***p < 0.001, ****p < 0.0001.

Figure S3 Effects of MEIS1 inhibition in vivo on cardiac structure and function. A–C) Gross anatomy assessment. A) Heart weight, B) Heart/body weight ratio, C) Heart weight/tibia length ratio. D–G) Echocardiography to evaluate left ventricular (LV) dimensions and function. D) Ejection fraction, E) LV diameter, F) Interventricular septum thickness, and G) Posterior wall thickness. H–M) ECG parameters. H) Heart rate, I) P wave duration, J) PR interval, K) QRS duration, L) QT interval, and M) corrected QT using Bazett’s formula (QTc). n = 5.

## Figures and Tables

**Table 1:** Primers used in the study.

Name	Primer (5′-3′)
Sox2-F	TGATGGAGACGGAGCTGAA
Sox2-R	GGGCTGTTTTTCTGGTTGC
Oct4-F	TCGAGAACCGAGTGAGAGG
Oct4-R	GAACCACACTCGGACCACA
Klf4-F	CGATCAGATGCAGCCGCAAGTC
Klf4-R	TGTGTAAGGCGAGGTGGTCCGA
Brachurty-F	GTGCTGTCCCAGGTGGCTTA
Brachurty-R	CCTTAACAGCTCAACTCTAA
Tert-F	GACGTGGAAGATGAGCGTG
Tert-R	GAGGACGTACACACTCATC
ISL1-F	AAACAGGAGCTCCAGCAAAA
ISL1-R	AAAGGACTCTTTCAGCCAAG
Nkx2.5-F	CTTCAAGCCAGAGGCCTACG
Nkx2.5-R	CCGCCTCTGTCTTCTCCAGC
TP53-F	ATTTGCGTGTGGAGTATTTGG
TP53-R	CCAGTAGATTACCACTGGAG
TnnT2-F	GGCAGCGGAAGAGGATGCTGAA
TnnT2-R	GAGGCACCAAGTTGGGCATGAACGA
COL1A1-F	CACACGTCTCGGTCATGGTA
COL1A1-R	AAGAGGAAGGCCAAGTCGAG
Cdh1(E-Cad)-F	AAAGGCCCATTTCCTAAAAACCT
Cdh1(E-Cad)-R	TGCGTTCTCTATCCAGAGGCT
Meis1-F	GGACAACAGCAGTGAGCAAG
Meis1-R	CACGCTTTTTGTGACGCTT
Meis2-F	GAAAAGGTCCACGAACTGTGC
Meis2-R	CTTTCATCAATGACGAGGTCGAT
GATA4-F	GACGGGTCACTATCTGTGCAA
GATA4-R	AGACATCGCACTGACTGAGAAC
Tbxt-F	TGACTGGCCTTAATCCCAAA
Tbxt-R	ACAAGTTGTCGCATCCAGTG
GAPDH-F	AATGAAGGGGTCATTGATGG
GAPDH-R	AAGGTGAAGGTCGGAGTCAA

**Table 2:** Primers for cardiac tissue assessment.

Name	Primer (5′-3′)
P15(CDK2NB)-F	CAGTTGGGTTCTGCTCCGT
P15(CDK2NB)-R	AGATCCCAACGCCCTGAAC
P16(CDKN2A)-F	GGGTTTCGCCCAACGCCCCGA
P16(CDKN2A)-R	TGCAGCACCACCAGCGTGTCC
P18(CDK2NC)-F	CTCCGGATTTCCAAGTTTCA
P18(CDK2NC)-R	GGGGGACCTAGAGCAACTTAC
P19(CDKN2D)-F	TCAGGAGCTCCAAAGCAACT
P19(CDKN2D)-R	TTCTTCATCGGGAGCTGGT
P19-ARF-F	GTTTTCTTGGTGAAGTTCGTGC
P19-ARF-R	TCATCACCTGGTCCAGGATTC
P21(CDKN1A)-F	ATCACCAGGATTGGACATGG
P21(CDKN1A)-R	CGGTGTCAGAGTCTAGGGGA
P27(CDKN1B)-F	GGGGAACCGTCTGAAACATT
P27(CDKN1B)-R	AGTGTCCAGGGATGAGGAAG
P57(CDKN1C)-F	TTCTCCTGCGCAGTTCTCTT
P57(CDKN1C)-R	CTGAAGGACCAGCCTCTCTC
Hif-1α-F	CGGCGAGAACGAGAAGAA
Hif-1α-R	AAACTTCAGACTCTTTGCTTCG
Hif-2α(EPAS1)-F	ATCACGGGATTTCTCCTTCC
Hif-2α(EPAS1)-R	GGTTAAGGAACCCAGGTGCT
Hif-3α-F	TGTGAACTTCATGTCCAGGC
Hif-3α-R	GCAATGCCTGGTGCTTATCT
Meis1-F	GTTGTCCAAGCCATCACCTT
Meis1-R	ATCCACTCGTTCAGGAGGAA
GAPDH-F	GAACCCTAAGGCCAACCGT
GAPDH-R	ACCGCTCGTTGCCAATAGTGATG

## References

[b1-tjb-48-06-414] Aksoz M Turan RF Albayrak E Kocabas F 2018 Emerging roles of Meis1 in cardiac regeneration, stem cells, and cancer Current Drug Targets 19 2 181 190 10.2174/1389450118666170724165514 28745213

[b2-tjb-48-06-414] Alam P Haile B Arif M Pandey R Rokvic M 2019 Inhibition of senescence-associated genes *Rb1* and *Meis2* in adult cardiomyocytes results in cell cycle reentry and cardiac repair post-myocardial infarction Journal of the American Heart Association 8 15 e012089 10.1161/JAHA.119.012089 PMC6761626 31315484

[b3-tjb-48-06-414] Arata Y Kouike H Zhang Y Herman MA Okano H 2006 Wnt signaling and a Hox protein cooperatively regulate psa-3/Meis to determine daughter cell fate after asymmetric cell division in C. elegans Developmental Cell 11 1 105 115 10.1016/j.devcel.2006.04.020 16824957

[b4-tjb-48-06-414] Arkin MR Wells JA 2004 Small-molecule inhibitors of protein-protein interactions: progressing towards the dream Nature Reviews Drug Discovery 3 4 301 317 10.1038/nrd1343 15060526

[b5-tjb-48-06-414] Ba L Gao J Chen Y Qi H Dong C 2019 Allicin attenuates pathological cardiac hypertrophy by inhibiting autophagy via activation of PI3K/Akt/mTOR and MAPK/ERK/mTOR signaling pathways Phytomedicine 58 152765 10.1016/j.phymed.2018.11.025 31005720

[b6-tjb-48-06-414] Bai Y Chen Q Sun YP Wang X Lv L 2017 Sulforaphane protection against the development of doxorubicin-induced chronic heart failure is associated with Nrf2 upregulation Cardiovascular Therapeutics 35 10.1111/1755-5922.12277 28636290

[b7-tjb-48-06-414] Becatti M Taddei N Cecchi C Nassi N Fiorillo C 2012 SIRT1 modulates MAPK pathways in ischemic-reperfused cardiomyocytes Cellular and Molecular Life Sciences 69 13 2245 2260 10.1007/s00018-012-0925-5 22311064 PMC11114949

[b8-tjb-48-06-414] Bhanvadia RR VanOpstall C Brechka H Barashi NS Gillard M 2018 MEIS1 and MEIS2 expression and prostate cancer progression: a role for HOXB13 binding partners in metastatic disease Clinical Cancer Research 24 15 3668 3680 10.1158/1078-0432.CCR-17-3673 29716922 PMC6082699

[b9-tjb-48-06-414] Brooks G Poolman RA Li JM 1998 Arresting developments in the cardiac myocyte cell cycle: role of cyclin-dependent kinase inhibitors Cardiovascular Research 39 301 311 10.1016/S0008-6363(98)00125-4 9798515

[b10-tjb-48-06-414] Brooks G Poolman RA McGill CJ Li JM 1997 Expression and activities of cyclins and cyclin-dependent kinases in developing rat ventricular myocytes Journal of Molecular and Cellular Cardiology 29 2261 2271 10.1006/jmcc.1997.0471 9281457

[b11-tjb-48-06-414] Burridge PW Matsa E Shukla P Lin ZC Cui B 2014 Chemically defined generation of human cardiomyocytes Nature Methods 11 8 855 10.1038/nmeth.2999 24930130 PMC4169698

[b12-tjb-48-06-414] Burstin J Bachhuber F Paul M Schmid RM Rustgi AK 2017 The TALE homeodomain transcription factor MEIS1 activates the pro-metastatic melanoma cell adhesion molecule Mcam to promote migration of pancreatic cancer cells Molecular Carcinogenesis 56 3 936 944 10.1002/mc.22547 27583552

[b13-tjb-48-06-414] Cabeza L Ortiz R Arias JL Prados J Ruiz Martinez MA 2015 Enhanced antitumor activity of doxorubicin in breast cancer through the use of poly(butylcyanoacrylate) nanoparticles International Journal of Nanomedicine 10 1291 1306 10.2147/IJN.S74378 25709449 PMC4335619

[b14-tjb-48-06-414] Chang CP Jacobs Y Nakamura T Jenkins NA Copeland NG 1997 Meis proteins are major in vivo DNA binding partners for wild-type but not chimeric Pbx proteins Molecular and Cellular Biology 17 10 5679 5687 10.1128/MCB.17.10.5679 9315626 PMC232416

[b15-tjb-48-06-414] Chen L Byer SH Holder R Wu L Burkey K 2023 Wnt10b protects cardiomyocytes against doxorubicin-induced cell death via MAPK modulation PLoS One 18 10 e0277747 10.1371/journal.pone.0277747 37856516 PMC10586692

[b16-tjb-48-06-414] Choi WY Gemberling M Wang J Holdway JE Shen MC 2013 In vivo monitoring of cardiomyocyte proliferation to identify chemical modifiers of heart regeneration Development 140 3 660 666 10.1242/dev.088526 23293297 PMC3561784

[b17-tjb-48-06-414] Delgado I Giovinazzo G Temiño S Gauthier Y Balsalobre A 2021 Control of mouse limb initiation and antero-posterior patterning by Meis transcription factors Nature Communications 12 3086 10.1038/s41467-021-23373-9 PMC8149412 34035267

[b18-tjb-48-06-414] Dibner C Elias S Frank D 2001 Meis3 protein activity is required for proper hindbrain patterning in Xenopus laevis embryos Development 128 3415 3426 10.1242/dev.128.18.3415 11566848

[b19-tjb-48-06-414] Doser TA Turdi S Thomas DP Epstein PN Li SY 2009 Transgenic overexpression of aldehyde dehydrogenase-2 rescues chronic alcohol intake-induced myocardial hypertrophy and contractile dysfunction Circulation 119 1941 1949 10.1161/CIRCULATIONAHA.108.823799 19332462 PMC2740924

[b20-tjb-48-06-414] Fabik J Kovacova K Kozmik Z Machon O 2020 Neural crest cells require Meis2 for patterning the mandibular arch via the Sonic hedgehog pathway Biology Open 9 6 bio052043 10.1242/bio.052043 32616504 PMC7331463

[b21-tjb-48-06-414] Ferreira HJ Heyn H Vizoso M Moutinho C Vidal E 2016 DNMT3A mutations mediate the epigenetic reactivation of the leukemogenic factor MEIS1 in acute myeloid leukemia Oncogene 35 3079 3082 10.1038/onc.2015.359 26434589 PMC4705435

[b22-tjb-48-06-414] Girgin B Kocabaş F 2023 Newly developed MEIS inhibitor selectively blocks MEISHigh prostate cancer growth and induces apoptosis Gene 871 147425 10.1016/j.gene.2023.147425 37044182

[b23-tjb-48-06-414] Girgin B Karadağ-Alpaslan M Kocabaş F 2020 Oncogenic and tumor suppressor function of MEIS and associated factors Turkish Journal of Biology 44 6 328 355 10.3906/biy-2006-25 33402862 PMC7759197

[b24-tjb-48-06-414] Golan-Lagziel T Lewis YE Shkedi O Douvdevany G Caspi LH 2018 Analysis of rat cardiac myocytes and fibroblasts identifies combinatorial enhancer organization and transcription factor families Journal of Molecular and Cellular Cardiology 116 91 105 10.1016/j.yjmcc.2018.02.003 29421235

[b25-tjb-48-06-414] Guo D Cheng L Shen Y Li W Li Q 2020 6-Bromoindirubin-3′-oxime (6BIO) prevents myocardium from aging by inducing autophagy Aging 12 24 26047 26062 10.18632/aging.202253 33401248 PMC7803501

[b26-tjb-48-06-414] Hegyi B Bers DM Bossuyt J 2019 CaMKII signaling in heart diseases: emerging role in diabetic cardiomyopathy Journal of Molecular and Cellular Cardiology 127 246 259 10.1016/j.yjmcc.2019.01.001 30633874

[b27-tjb-48-06-414] Helmstadter KG Ljubojevic-Holzer S Wood BM Taheri KD Sedej S 2021 CaMKII and PKA-dependent phosphorylation co-regulate nuclear localization of HDAC4 in adult cardiomyocytes Basic Research in Cardiology 116 1 11 10.1007/s00395-021-00850-2 33590335 PMC7884572

[b28-tjb-48-06-414] Henriksen PA 2018 Anthracycline cardiotoxicity: an update on mechanisms, monitoring, and prevention Heart 104 971 977 10.1136/heartjnl-2017-312103 29217634

[b29-tjb-48-06-414] Herrmann J 2020 Adverse cardiac effects of cancer therapies: cardiotoxicity and arrhythmia Nature Reviews Cardiology 17 8 474 502 10.1038/s41569-020-0348-1 32231332 PMC8782611

[b30-tjb-48-06-414] Hesselbarth R Esser TU Roshanbinfar K Schrüfer S Schubert DW 2021 CHIR99021 promotes hiPSC-derived cardiomyocyte proliferation in engineered 3D microtissues Advanced Healthcare Materials 10 20 e2100926 10.1002/adhm.202100926 34499814 PMC11468594

[b31-tjb-48-06-414] Imamura T Morimoto A Takanashi M Hibi S Sugimoto T 2002 Frequent co-expression of HoxA9 and Meis1 genes in infant acute lymphoblastic leukaemia with MLL rearrangement British Journal of Haematology 119 119 121 10.1046/j.1365-2141.2002.03803.x 12358913

[b32-tjb-48-06-414] Jiang M Xu S Bai M Zhang A 2021 The emerging role of MEIS1 in cell proliferation and differentiation American Journal of Physiology -Cell Physiology 320 3 C264 C269 10.1152/ajpcell.00422.2020 33296285

[b33-tjb-48-06-414] Karakikes I Ameen M Termglinchan V Wu JC 2015 Human induced pluripotent stem cell-derived cardiomyocytes: insights into molecular, cellular, and functional phenotypes Circulation Research 117 1 80 88 10.1161/CIRCRESAHA.117.305365 26089365 PMC4546707

[b34-tjb-48-06-414] Khairnar SI Kulkarni YA Singh K 2022 Cardiotoxicity linked to anticancer agents and cardioprotective strategy Archives of Pharmacal Research 45 10 704 730 10.1007/s12272-022-01411-4 36306018

[b35-tjb-48-06-414] Kim YS Jeong HY Kim AR Kim WH Cho H 2016 Natural product derivative BIO promotes recovery after myocardial infarction via unique modulation of the cardiac microenvironment Scientific Reports 6 30726 10.1038/srep30726 27510556 PMC4980696

[b36-tjb-48-06-414] Kocabas F Zheng J Thet S Sadek H 2012 Meis1 regulates the metabolic phenotype and oxidant defense of hematopoietic stem cells Blood 120 25 10.1182/blood-2012-05-432260 PMC3525021 22995899

[b37-tjb-48-06-414] Kubin T Cetinkaya A Kubin N Bramlage P Sen-Hild B 2020 The MEK/ERK module is reprogrammed in remodeling adult cardiomyocytes International Journal of Molecular Sciences 21 17 6348 10.3390/ijms21176348 32882982 PMC7503571

[b38-tjb-48-06-414] Laurent A Bihan R Omilli F Deschamps S Pellerin I 2008 PBX proteins: much more than Hox cofactors International Journal of Developmental Biology 52 1 9 20 10.1387/ijdb.072304al 18033668

[b39-tjb-48-06-414] Li J Zhu D Hu S Nie Y 2022 CRISPR-CasRx knock-in mice for RNA degradation Science China Life Sciences 65 11 2248 2256 10.1007/s11427-021-2059-5 35412223

[b40-tjb-48-06-414] Lindgren IM Drake RR Chattergoon NN Thornburg KL 2019 Down-regulation of MEIS1 promotes the maturation of oxidative phosphorylation in perinatal cardiomyocytes FASEB Journal 33 6 7417 7426 10.1096/fj.201801330RR 30884246 PMC6529342

[b41-tjb-48-06-414] Liu H Barnes J Pedrosa E Herman NS Salas F 2020 Transcriptome analysis of neural progenitor cells derived from Lowe syndrome induced pluripotent stem cells: identification of candidate genes for the neurodevelopmental and eye manifestations Journal of Neurodevelopmental Disorders 12 14 10.1186/s11689-020-09317-2 32393163 PMC7212686

[b42-tjb-48-06-414] Liu Y Li J Xu N Yu H Wang N 2022 Transcription factor Meis1 acts as a new regulator of ischemic arrhythmias in mice Journal of Advanced Research 39 275 289 10.1016/j.jare.2021.11.004 35777912 PMC9263651

[b43-tjb-48-06-414] Machon O Masek J Machonova O Krauss S Kozmik Z 2015 Meis2 is essential for cranial and cardiac neural crest development BMC Developmental Biology 15 40 10.1186/s12861-015-0093-6 26545946 PMC4636814

[b44-tjb-48-06-414] Mahmoud AI Kocabas F Muralidhar SA Kimura W Koura AS 2013 Meis1 regulates postnatal cardiomyocyte cell cycle arrest Nature 497 10.1038/nature12054 PMC4159712 23594737

[b45-tjb-48-06-414] Mallo M Alonso CR 2013 The regulation of Hox gene expression during animal development Development 140 19 3951 3963 10.1242/dev.068346 24046316

[b46-tjb-48-06-414] Meijers WC de Boer RA 2019 Common risk factors for heart failure and cancer Cardiovascular Research 115 5 844 853 10.1093/cvr/cvz035 30715247 PMC6452432

[b47-tjb-48-06-414] Meriç N Albayrak E Kocabaş F Gulbas Z 2023 MEIS inhibitors reduce the viability of primary leukemia cells and stem cells by inducing apoptosis Leukemia and Lymphoma 10.1080/10428194.2023.2275532 37902585

[b48-tjb-48-06-414] Meriç N Kocabas F 2022 The historical relationship between Meis1 and leukemia Advances in Experimental Medicine and Biology 1387 127 144 10.1007/5584_2021_705 35304708

[b49-tjb-48-06-414] Mercurio V Pirozzi F Lazzarini E Marone G Rizzo P 2016 Models of heart failure based on the cardiotoxicity of anticancer drugs Journal of Cardiac Failure 22 6 449 458 10.1016/j.cardfail.2016.04.008 27103426

[b50-tjb-48-06-414] Miller ME Rosten P Lemieux ME Lai C Humphries RK 2016 Meis1 is required for adult mouse erythropoiesis, megakaryopoiesis, and hematopoietic stem cell expansion PLoS One 11 3 e0151584 10.1371/journal.pone.0151584 26986211 PMC4795694

[b51-tjb-48-06-414] Morelli MB Bongiovanni C Da Pra S Miano C Sacchi F 2022 Cardiotoxicity of anticancer drugs: molecular mechanisms and strategies for cardioprotection Frontiers in Cardiovascular Medicine 9 847012 10.3389/fcvm.2022.847012 35497981 PMC9051244

[b52-tjb-48-06-414] Moskow JJ Bullrich F Huebner K Daar IO Buchberg AM 1995 *Meis1*, a *PBX1*-related homeobox gene involved in myeloid leukemia in BXH-2 mice Molecular and Cellular Biology 15 10 5434 5443 10.1128/MCB.15.10.5434 7565694 PMC230793

[b53-tjb-48-06-414] Paul S Zhang X He JQ 2019 Homeobox gene Meis1 modulates cardiovascular regeneration Seminars in Cell & Developmental Biology 10.1016/j.semcdb.2019.10.003 PMC7839269 31623926

[b54-tjb-48-06-414] Perea-Gil I Seeger T Bruyneel AAN Termglinchan V Mercola M 2022 Serine biosynthesis as a novel therapeutic target for dilated cardiomyopathy European Heart Journal 43 36 3477 3489 10.1093/eurheartj/ehac305 35728000 PMC9794189

[b55-tjb-48-06-414] Perez-Roger I Kim SH Griffiths B Sewing A Land H 1999 Cyclins D1 and D2 mediate Myc-induced proliferation via sequestration of p27^Kip1^ and p21^Cip1^ The EMBO Journal 18 5310 5320 10.1093/emboj/18.19.5310 10508164 PMC1171601

[b56-tjb-48-06-414] Poolman RA Brooks G 1998 Expressions and activities of cell cycle regulatory molecules during the transition from myocyte hyperplasia to hypertrophy Journal of Molecular and Cellular Cardiology 30 2121 2135 10.1006/jmcc.1998.0808 9799664

[b57-tjb-48-06-414] Poolman RA Gilchrist R Brooks G 1998 Cell cycle profiles and expressions of p21^CIP1^ and P27^KIP1^ during myocyte development International Journal of Cardiology 67 133 142 10.1016/S0167-5273(98)00320-9 9891946

[b58-tjb-48-06-414] Poolman RA Li JM Durand B Brooks G 1999 Altered expression of cell cycle proteins and prolonged duration of cardiac myocyte hyperplasia in p27KIP1 knockout mice Circulation Research 85 117 126 10.1161/01.RES.85.2.117 10417393

[b59-tjb-48-06-414] Quaranta R Rao J Piccini I Araúzo-Bravo MJ Greber B 2018 Revised roles of ISL1 in a hES cell-based model of human heart chamber specification eLife 7 e31706 10.7554/eLife.31706 29337667 PMC5770158

[b60-tjb-48-06-414] Rastegar-Pouyani S Khazaei N Wee P Yaqubi M Mohammadnia A 2017 Meta-analysis of transcriptome regulation during induction to cardiac myocyte fate from mouse and human fibroblasts Journal of Cellular Physiology 232 8 2053 2062 10.1002/jcp.25580 27579918

[b61-tjb-48-06-414] Sachinidis A 2020 Cardiotoxicity and heart failure: lessons from human-induced pluripotent stem cell-derived cardiomyocytes and anticancer drugs Cells 9 4 1001 10.3390/cells9041001 32316481 PMC7226145

[b62-tjb-48-06-414] Sun C Liu H Si K Wu Y Zhao K 2019 Meis2 represses the osteoblastic transdifferentiation of aortic valve interstitial cells through the Notch1/Twist1 pathway Biochemical and Biophysical Research Communications 509 2 455 461 10.1016/j.bbrc.2018.12.040 30594396

[b63-tjb-48-06-414] Turan RD Kocabas F 2020 Development of small molecule MEIS inhibitors that modulate HSC activity Scientific Reports 10 1 7994 10.1038/s41598-020-64888-3 32409701 PMC7224207

[b64-tjb-48-06-414] VanOpstall C Perike S Brechka H Gillard M Lamperis S 2020 MEIS-mediated suppression of human prostate cancer growth and metastasis through HOXB13-dependent regulation of proteoglycans eLife 9e53600 10.7554/eLife.53600 PMC7371429 32553107

[b65-tjb-48-06-414] Walasek MA van Os R de Haan G 2012 Hematopoietic stem cell expansion: challenges and opportunities Annals of the New York Academy of Sciences 1266 138 150 10.1111/j.1749-6632.2012.06549.x 22901265

[b66-tjb-48-06-414] Wang SH Cui LG Su XL Komal S Ni RC 2022a GSK-3β-mediated activation of NLRP3 inflammasome leads to pyroptosis and apoptosis of rat cardiomyocytes and fibroblasts European Journal of Pharmacology 920 174830 10.1016/j.ejphar.2022.174830 35182545

[b67-tjb-48-06-414] Wang X Ghareeb WM 2019 Hypermethylated and downregulated MEIS2 are involved in stemness properties and oxaliplatin-based chemotherapy resistance of colorectal cancer Journal of Cellular Physiology 234 10 18180 18191 10.1002/jcp.28451 30859572

[b68-tjb-48-06-414] Wang Z Yao M Jiang L Wang L Yang Y 2022b Dexmedetomidine attenuates myocardial ischemia/reperfusion-induced ferroptosis via AMPK/GSK-3β/Nrf2 axis Biomedical Pharmacotherapy 154 113572 10.1016/j.biopha.2022.113572 35988428

[b69-tjb-48-06-414] Zhang J Liang R Wang K Zhang W Zhang M 2022 Novel CaMKII-δ inhibitor Hesperadin exerts dual functions to ameliorate cardiac ischemia/reperfusion injury and inhibit tumor growth Circulation 145 15 1154 1168 10.1161/CIRCULATIONAHA.121.055920 35317609

[b70-tjb-48-06-414] Zhang X Friedman A Heaney S Purcell P Maas RL 2002 Meis homeoproteins directly regulate Pax6 during vertebrate lens morphogenesis Genes & Development 16 2097 2107 10.1101/gad.1007602 12183364 PMC186446

[b71-tjb-48-06-414] Zhang Y Si Y Ma N 2016 Meis1 promotes poly (rC)-binding protein 2 expression and inhibits angiotensin II-induced cardiomyocyte hypertrophy IUBMB Life 68 13 22 10.1002/iub.1456 26597775

