# Supplementary figures and images for: Mechanistic insights into cardiac regeneration and protection through MEIS inhibition
Source: Turk J Biol. 2024 Oct 30;48(6):414–31. doi: 10.55730/1300-0152.2716 (PMC11698199; doi:10.55730/1300-0152.2716)

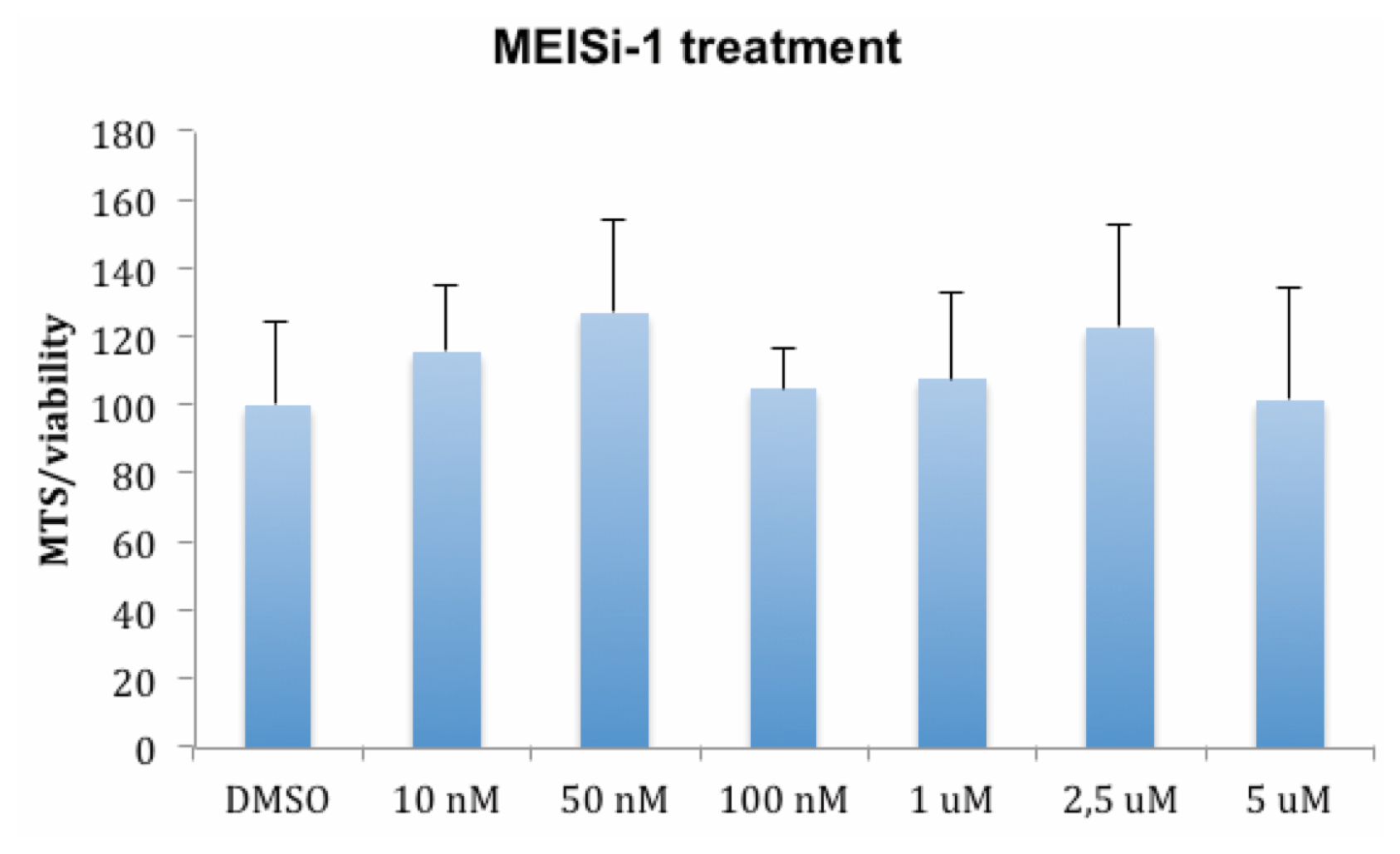

Supplement: Figure S1 (related to Figure 3) — Analysis of iPSC viability and mitochondrial activity via MTS tetrazolium assay post-MEISi-1 treatments. Increasing doses of MEISi-1 (10 nM up to 5 μM) were assessed in hiPSCs for cell viability. [file tjb-48-06-414s1.tif]

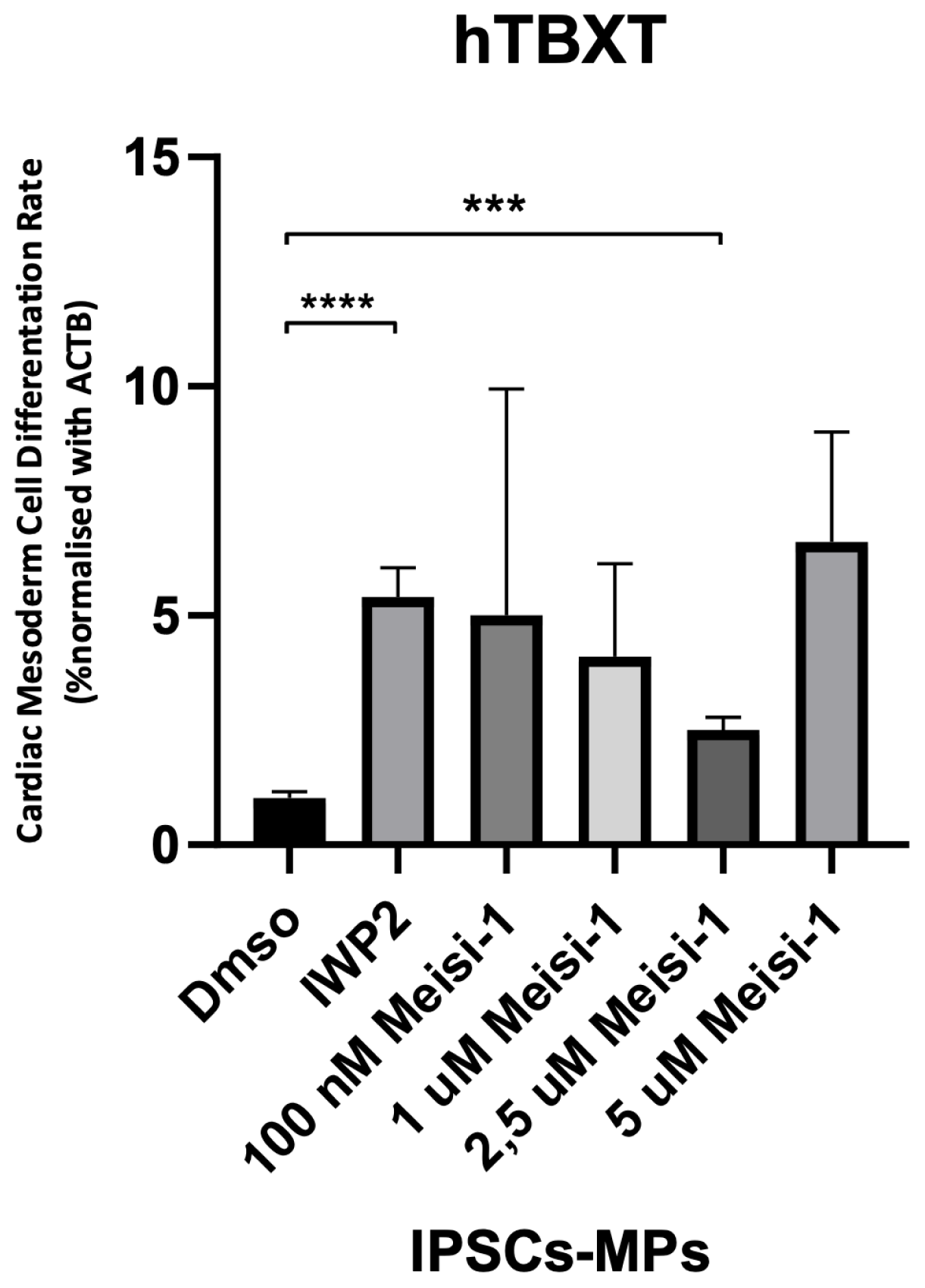

Supplement: Figure S2 (related to Figure 5) — Effect of short-term MEIS1 inhibition in TBXT expression during IPSC differentiation. n = 3, ***p < 0.001, ****p < 0.0001. [file tjb-48-06-414s2.tif]

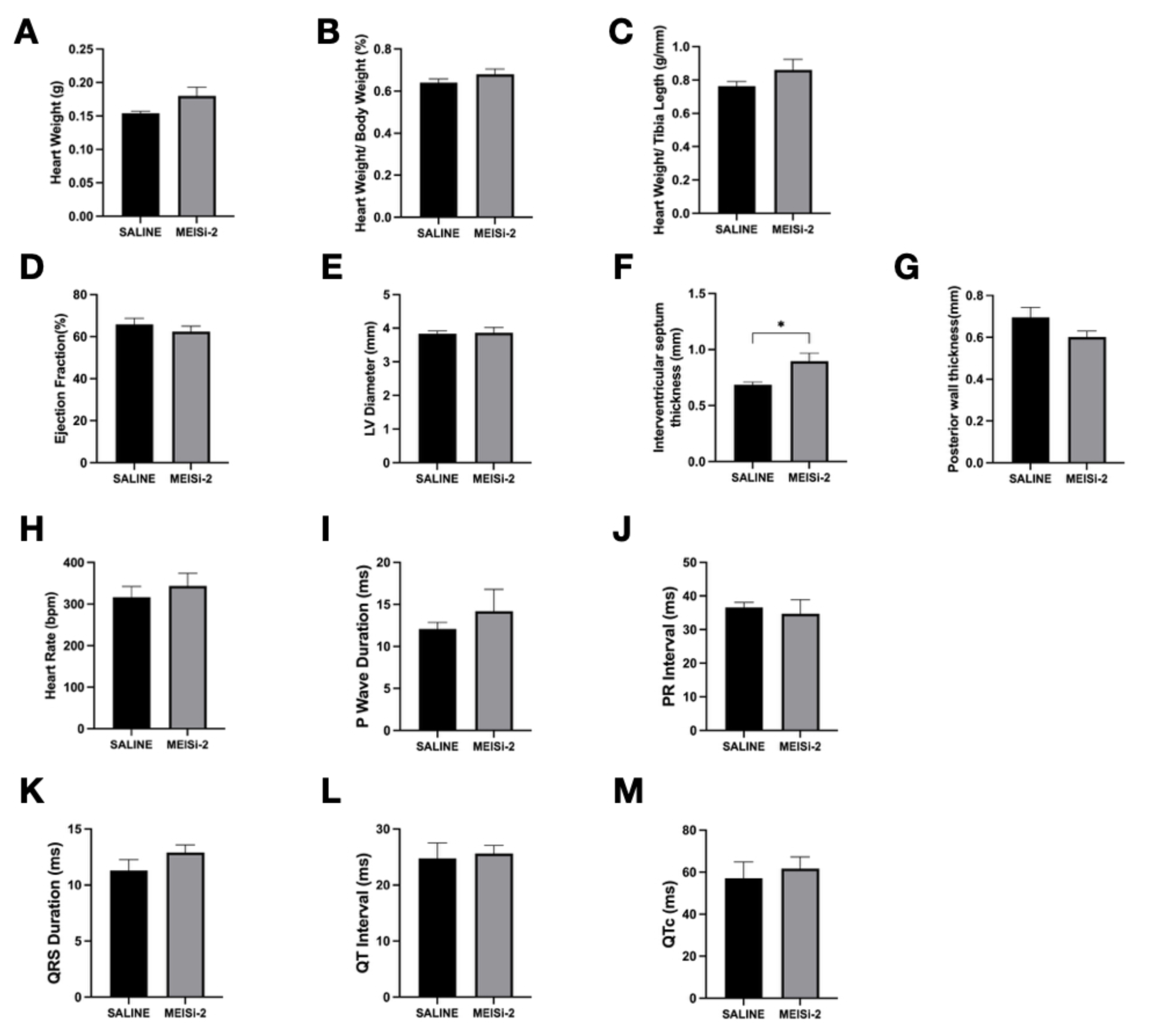

Supplement: Figure S3 — Effects of MEIS1 inhibition in vivo on cardiac structure and function. A–C) Gross anatomy assessment. A) Heart weight, B) Heart/body weight ratio, C) Heart weight/tibia length ratio. D–G) Echocardiography to evaluate left ventricular (LV) dimensions and function. D) Ejection fraction, E) LV diameter, F) Interventricular septum thickness, and G) Posterior wall thickness. H–M) ECG parameters. H) Heart rate, I) P wave duration, J) PR interval, K) QRS duration, L) QT interval, and M) corrected QT using Bazett’s formula (QTc). n = 5. [file tjb-48-06-414s3.tif]
